# A Scalar Poincaré Map for Anti-phase Bursting in Coupled Inhibitory Neurons With Synaptic Depression

**DOI:** 10.3389/fams.2022.822782

**Published:** 2022-06-02

**Authors:** Mark Olenik, Conor Houghton

**Affiliations:** 1 School of Biological Sciences, Faculty of Life Sciences, University of Bristol, Bristol, United Kingdom; 2 School of Computer Science, Electrical and Electronic Engineering, and Engineering Mathematics, Faculty of Engineering, University of Bristol, Bristol, United Kingdom

**Keywords:** Poincaré map, neuronal bursting, dynamical system (DS), synaptic depression, central pattern generator

## Abstract

Short-term synaptic plasticity is found in many areas of the central nervous system. In the inhibitory half-center central pattern generators involved in locomotion, synaptic depression is believed to act as a burst termination mechanism, allowing networks to generate anti-phase bursting patterns of varying periods. To better understand burst generation in these central pattern generators, we study a minimal network of two neurons coupled through depressing synapses. Depending on the strength of the synaptic conductance between the two neurons, this network can produce symmetric *n* : *n* anti-phase bursts, where neurons fire *n* spikes in alternation, with the period of such solutions increasing with the strength of the synaptic conductance. Relying on the timescale disparity in the model, we reduce the eight-dimensional network equations to a fully-explicit scalar Poincaré burst map. This map tracks the state of synaptic depression from one burst to the next and captures the complex bursting dynamics of the network. Fixed points of this map are associated with stable burst solutions of the full network model, and are created through fold bifurcations of maps. We derive conditions that predict the bifurcations between *n* : *n* and (*n* + 1) : (*n* + 1) solutions, producing a full bifurcation diagram of the burst cycle period. Predictions of the Poincaré map fit excellently with numerical simulations of the full network model and allow the study of parameter sensitivity for rhythm generation.

## 1 Introduction

Short-term synaptic plasticity may have a role in burst activity in central pattern generators (CPGs). Short-term synaptic depression is commonly found in neuronal networks involved in the generation of rhythmic movements, such as in the pyloric CPG of the spiny lobster [1, 2], or in the lumbosacral cord of the chick embryo [3]. Synaptic depression modulates the strength of synapses in response to changes to the presynaptic firing frequency. At a high neuronal firing frequency, depression weakens the strength of synapses and therefore reduces the magnitude of the postsynaptic response. At low firing frequency, it allows sufficient time for the synapse to recover from depression between spikes, leading to a stronger postsynaptic response. In reciprocal networks, synaptic depression has been shown to act as a “switch,” giving rise to a wide range of network dynamics such as synchronous and multi-stable rhythms, as well as fine tuning the frequency of network oscillations [4–6].

Brown [7] pioneered the idea that synaptic depression acts as a burst termination mechanism in CPGs composed of reciprocally inhibitory neurons and involved in rhythm generation of locomotion. When one side is firing during a burst the other, antagonistic side, is prevented from firing by synaptic inhibition. However, the weakening of inhibition as a result of synaptic depression eventually releases the antagonistic side so that it starts firing, terminating the burst on the side that had originally been firing. This rhythmogenesis hypothesis has been considered one of a handful of standard mechanisms for generating locomotion rhythms in vertebrates [8–10]. It has been proposed as an explanation of the antiphase burst rhythm in struggling in *Xenopus* tadpoles [11].

Bose and Booth [6] investigated burst generation in a generic half-center CPG that consists of two identical, tonically active Morris-Lecar [12] neurons coupled through inhibitory depressing synapses. Numerical simulations showed that when the reciprocal synaptic conductance between the two neurons is varied, the network produces symmetric *n* : *n* anti-phase bursts, with stronger synaptic coupling leading to longer bursts. They used methods from geometric singular perturbation theory to separate the timescales of the fast membrane, and the slow synaptic dynamics of the network to derive one-dimensional conditions necessary for the existence of stable *n* : *n* solutions (for *n* ≤ 2). According to these conditions the type of firing pattern largely depends on the slow depression dynamics of the synapses between the two neurons, and can therefore be predicted by knowing the strengths of the synaptic conductances of the two synapses. Thus, the scalar conditions derived in Bose and Booth [6] provide a method to numerically identify the type of stable *n* : *n* pattern for any given value of the coupling strength and *n* ≤ 2. However, they do not predict the exact period of such solutions. Furthermore, while they provide good arguments for the validity of their reduction assumptions and the resulting scalar conditions, they do not verify them numerically.

Here we extend the previous analysis by providing a Poincaré map of the slow depression dynamics. This allows us not only to predict the types of stable *n* : *n* solutions the full network can produce, (for any *n*), but also to study how varying the coupling strength affects the period of such solutions. To do this, we build on, and numerically test, the assumptions on the fast-slow timescale disparity made in Bose and Booth [6]. We reduce the two-cell model to a scalar Poincaré map that tracks the evolution of the depression from the beginning of one burst to the beginning of the next burst. Stable fixed points of our map are associated with stable *n* : *n* burst solutions. Our map construction is motivated by the burst length map of a T-type calcium current, utilized by Matveev et al. [13], which approximates the anti-phase bursting dynamics of a network of two coupled Morris-Lecar neurons. In contrast to our model, the network described in the Matveev et al. [13] paper does not contain short-term synaptic depression, and burst termination is instead accomplished through the dynamics of a slow T-type calcium current.

The Poincaré map derived here replicates the results from numerical simulations of the full two-cell ODE system: Given the strength of maximum conductance between the two neurons, fixed points of our map predict the type and period of *n* : *n* patterns, the switch between burst solutions of different periods, as well as the occurrence of co-existent solutions. In addition to proving the existence and stability of fixed points, our map shows that fixed points are created via a fold bifurcation of maps. Finally, we use our map to derive algebraic conditions that allow us to predict parameter values of the maximum conductance at which *n* : *n* solutions bifurcate to (*n* + 1) : (*n* + 1) solutions, and vice versa. Because our map is fully explicit, it lays the framework for studying the effects of other model parameters on network dynamics without the need to run expensive numerical integrations of the ODEs.

This paper is organized as follows. First, we introduce the network of two neurons, and describe the properties of single cell and synapse dynamics. We use numerical simulations of the network to provide an intuition for the range of possible burst dynamics the system can produce. Next, we state and justify the simplifying assumptions that are necessary for the map construction. Finally, we analytically derive the first return map of the depression variable as well as the conditions that are required for stable *n* : *n* solutions. We end this work with a discussion.

## 2 Materials And Methods

We consider a pair of identical Morris-Lecar neurons [12], with parameters from Bose and Booth [6]. The Morris-Lecar model is a set of two first-order differential equations that describe the membrane dynamics of a spiking neuron. The depolarisation is modeled by an instantaneous calcium current, and the hyperpolarisation by a slow potassium current and a leak current. The membrane potential *v_i_* and potassium activation *w_i_* of neuron *i* (*i*, *j* = 1, 2) is described by:

(1) $${\dot{v}}_{i}=f(v_{i},w_{i})-\bar{g}s_{j}(v_{i}-v_{s}),$$

(2) $${\dot{w}}_{i}=h(v_{i},w_{i}).$$

Here *v_s_* is the inhibitory reversal potential, and $\bar{g}$ and *s_j_* are the maximal synaptic conductance and the synaptic gating, respectively, constituting the total inhibitory conductance $\bar{g}s_{j}$ from neuron *j* to neuron *i*. Function *f* (*v_i_, w_i_*) describes the membrane currents of a single cell:

(3) $$\begin{array}{c} f(v_{i},w_{i})=-g_{\text{Ca}}m_{\infty }(v_{i})(v_{i}-v_{\text{Ca}})-g_{\text{K}}w_{i}(v_{i}-v_{\text{K}})\\ -g_{\text{L}}(v_{i}-v_{\text{L}})+I. \end{array}$$

The currents include a constant current *I*, and three ionic currents: an instantaneous calcium current, a potassium current, and a leak current, with respective reversal potentials *v*_Ca_, *v*_K_, and *v*_L_, as well as maximum conductances *g*_Ca_, *g*_K_, and *g*_L_. The function *h*(*v_i_*, *w_i_*) models the kinetics of the potassium gating variable *w_i_*, and is given by

(4) $$h(v_{i},w_{i})=\frac{w_{\infty }(v_{i})-w_{i}}{\tau_{w}}.$$

The steady-state activation functions *m_∞_* and *w_∞_* as well as the default model parameters are described in the Supplementary Material 1.

The dynamics of the synaptic interactions between the neurons are governed by a synaptic gating variable *s_i_* and a depression variable *d_i_*:

(5) $${\dot{d}}_{i}=\{\begin{array}{ll} (1-d_{i})/\tau_{a} & \text{if}\;v_{i}<v_{\theta },\\ -d_{i}/\tau_{b} & \text{if}\;v_{i}>v_{\theta }, \end{array}$$

(6) $${\dot{s}}_{i}=\{\begin{array}{ll} -s_{i}/\tau_{\kappa } & \text{if}\;v_{i}<v_{\theta },\\ 0 & \text{if}\;v_{i}>v_{\theta }. \end{array}$$

Variable *d_i_* describes a firing rate dependent depletion mechanism that governs the amount of depression acting on the synapse. The model is agnostic with respect to the exact mechanism of this depletion, be it pre- or post-synaptic. When the voltage of cell *i* is above firing threshold (*v_i_* > *v_θ_*), variable *d_i_* decays with time constant *τ_b_*, and recovers with time constant *τ_a_* when voltage is below firing threshold (*v_i_* < *v_θ_*). Since the synaptic inhibition occurs on a much faster timescale than synaptic depression, we assume that *s_i_* is instantaneously reset to *d_i_* whenever *v_i_* increases above *v_θ_*, where it remains throughout *v_i_* > *v_θ_*. Whenever *v_i_* < *v_θ_*, the synaptic variable decays exponentially with time constant *τ_k_*. The equations for the depression model are identical to the Bose and Booth [14] model. These equations are a mathematically tractable simplification of the established phenomenological depression model previously described by Tsodyks and Markram [15].

**Figure 1:**
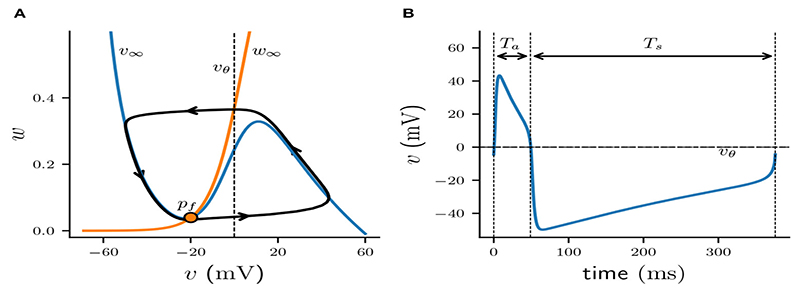
Periodic solution of ML model neuron. **(A)** Projection of limit cycle onto (*v, w*)-phase plane with v-nullcline (blue, *v*_∞_) and *w*-nullcline (orange, *w*_∞_). Unstable fixed point *p_f_* is indicated by an orange dot; firing threshold *v_θ_* is denoted by a dashed line. **(B)** Corresponding voltage trace *v*(*t*) of an action potential.

When the total inhibitory conductance $\bar{g}s_{j}$ is constant, the membrane dynamics are determined by the cubic *v*-nullcline *v_∞_*(*v_i_*) and the sigmoid *w*-nullcline *w*_∞_(*v_i_*), satisfying ${\dot{v}}_{i}=0$ and ${\dot{w}}_{i}=0,$ respectively. In case of no inhibition $(\bar{g}=0),$ the two curves intersect near the local minimum of *v_∞_* to the left of *v*_θ_ (commonly referred to as “left knee” of *v_∞_*), creating an unstable fixed point *p_f_* with a surrounding stable limit cycle of period *T* = *T_a_* + *T_s_* (Figure 1A). Here *T_a_* is the amount of time the membrane potential spends above firing threshold (*v_i_* > *v_θ_*), while *T_s_* is the time it spends below firing threshold (*v_i_* < *v_θ_*). Trajectories along that limit cycle have the familiar shape of the action potential (Figure 1B). Applying a constant nonzero inhibition, e.g., by letting *s_j_* = 1 and $\bar{g}>0,$ moves the cubic *v*_∞_ with the ensuing unstable fixed point down *w*_∞_ in the (*v_i_*, *w_i_*) -plane. When $\bar{g}$ is large enough, the fixed point moves past the left knee and becomes stable via a subcritical Andoronov-Hopf bifurcation, attracting all previously periodic trajectories. In the following section we will refer to the value of the total conductance $\bar{g}s_{j}$ at the bifurcation point as *g_bif_*.

The two-cell network model is numerically integrated using an adaptive step-size integrator for stiff differential equations implemented with XPPAUT [16] and controlled through the Python packages SciPy [17] and PyXPP [18]. The following mathematical analysis is performed on the equations of a single cell. Unless required for clarity, we will therefore omit the subscripts *i*, *j* from here on.

## 3 Results

### 3.1 Anti-phase Burst Solutions

**Figure 2:**
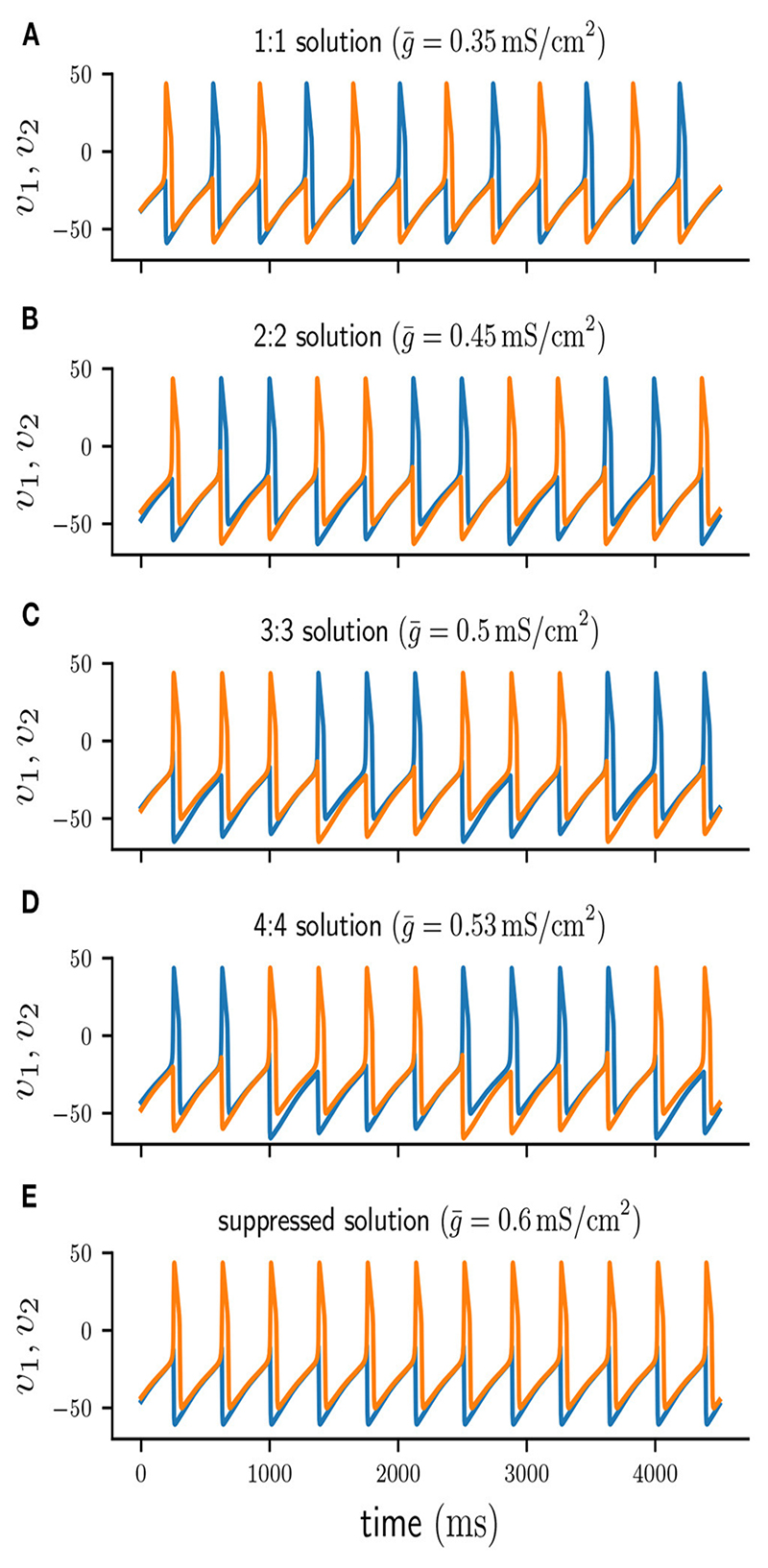
Voltage traces of cell 1 (blue) and cell 2 (orange) of numerically stable solutions. **(A–D)** 1 : 1,2: 2, 3 : 3, and 4 : 4 anti-phase solutions for increasing values of $\bar{g}.$ **(E)** Suppressed solution.

Short-term synaptic depression of inhibition in a half-center oscillator acts as a *burst termination* mechanism [7] and is known to produce *n* : *n* anti-phase burst solutions of varying period. Such *n* : *n* solutions consist of cells firing bursts of *n* spikes in alternation. Figure 2D shows the timecourse of a typical 4 : 4 burst. While one cell is firing a burst it provides an inhibitory conductance to the other cell, preventing it from firing.

Therefore, at any given moment one cell is spiking while the other is suppressed and does not spike. We will refer to the currently firing cell as “active” and we will call the suppressed cell “silent.” Additionally, we will distinguish between two phases of a *n* : *n* solution: We will refer to the time interval when a cell is firing as the “active phase,” and we will call the remaining duration of a cycle, when a cell is not firing, the “silent phase.”

With each action potential of the active cell, short-term depression leads to a decrease of *d*, and consequently of *s*. If *d* depresses faster at spike time than it can recover in the inter-spike-intervals (*ISIs*), the total synaptic conductance $\bar{g}s$ will eventually become sufficiently small to allow for the silent cell to be released [19, 20] and start firing, thus inhibiting the previously active cell. While a cell is silent its depression variable can recover. Once the silent cell becomes active again its synaptic inhibition will be sufficient to terminate the burst of the previously active cell and commence a new cycle. As previously demonstrated by Bose and Booth [6], in a two-cell reciprocally inhibitory network with synaptic depression the coupling strength $\bar{g}$ determines the type of *n* : *n* solution. Increasing $\bar{g}$ produces higher *n* : *n* burst solutions with more spikes per burst and a longer cycle period. Figure 2 shows numerically stable *n* : *n* solutions for varying values of $\bar{g}.$ For small values of $\bar{g}$ the network produces anti-phase spiking 1 : 1 solutions (Figure 2A). As $\bar{g}$ is increased the network generates solutions of increasing *n*, that is 2 : 2 (Figure 2B), 3 : 3 (Figure 2C), and 4 : 4 (Figure 2D). When $\bar{g}$ is sufficiently large (Figure 2E), one of the cells continuously spikes at its uncoupled period *T* while the other cell remains fully suppressed. Depending on the initial conditions either of the two cells can become the suppressed cell, which is why the suppressed solution is numerically bistable.

**Figure 3:**
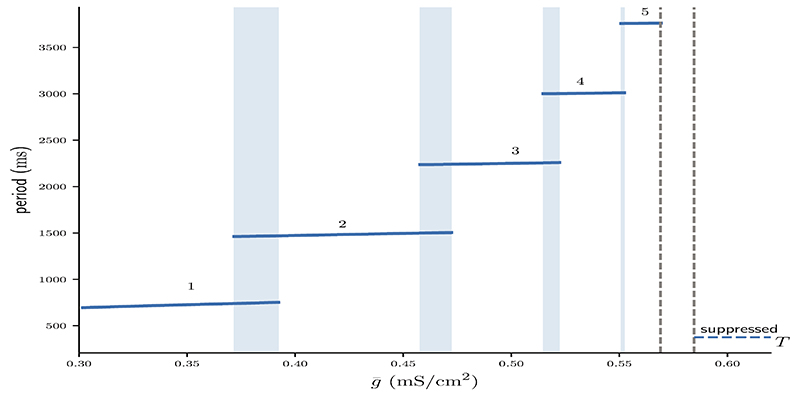
Numerically computed bifurcation diagram of the cycle period of stable *n* : *n* solutions for increasing coupling strength $\bar{g}.$. Regions of bistability are indicated by light blue vertical stripes. Dashed lines show the interval between the 5 : 5 and the suppressed solution, where higher period *n* : *n* solutions occur on increasingly smaller intervals of $\bar{g}.$

Branches of numerically stable *n* : *n* solutions and their associated limit cycle period for varying values of $\bar{g}$ are depicted in Figure 3 (see Supplementary Material 2 for algorithm description). Not only do higher *n* : *n* solutions branches require stronger coupling $\bar{g},$ but also within *n* : *n* branches the period increases with $\bar{g}.$ In line with Bose and Booth [6] we find small overlaps between solution branches indicating numerical bistability, for example such as between the 2 : 2 and 3 : 3 solution branches. Branches of higher *n* : *n* burst solutions occur on increasingly smaller intervals of $\bar{g},$ for instance is the $\bar{g}$ interval of the 5 : 5 branch shorter than that of the 4 : 4 branch and so on. The interval between the 5 : 5 branch and the suppressed solution (region between dotted lines in Figure 3) not only contains even higher numerically stable *n* : *n* solutions, such as 11 : 11 bursts, but also other non-symmetric *n* : *m* solutions as well as irregular, non-periodic solutions. However, the analysis in the following sections will only be concerned with the numerically stable and symmetric *n* : *n* solutions.

### 3.2 Mathematical Analysis of Two-Cell Network

The goal of the following mathematical analysis is to reduce the complexity of the eight-dimensional system to a more tractable problem. As we will explain, we do this by approximating the full dynamics by a reduced system that describes the evolution of the depression variable *d* of either of the two cells. We will construct the solution of *d* in a piecewise manner from one spike to the next, first during the active phase, and then during the silent phase. This construction will require two assumptions about the membrane and synaptic dynamics. The first assumption states that during a burst the active cell fires at its uncoupled period *T*, which simplifies the construction of the solution of *d*. The second assumption states that once the inhibitory conductance acting on the silent cell drops below a critical threshold, the cell is immediately released and fires. The second assumption is necessary to predict the release time of the silent cell, which allows us to model the recovery of *d* during the silent phase. In other words, the second assumption requires that the release of the silent cell from inhibition depends only on the timecourse of the inhibition, and not on the membrane dynamics of the silent cell. The approximate validity of both assumptions can be observed in coupled relaxation-oscillator types of neurons such as the Morris-Lecar model we use, and will be numerically verified below. Both assumptions were first used in Bose and Booth [6] to derive algebraic conditions that guarantee the periodicity of the depression variable for different *n* : *n* solutions. However, here we will use these assumptions to construct a Poincaré map of *d*, which will provide a geometric intuition for the dynamics of the full two-cell network and its dependence on model parameters.

Our first assumption about the model states that the active cell fires at its uncoupled period *T*, that is, during the active phase of a burst we have *ISI = T*. Solution profiles in Figure 2 suggest that the *ISI*s are indeed approximately constant. Numerically computing *ISIs* for all stable *n* : *n* solutions in Figure 3 reveals that *ISI*s differ by at most 1 *ms* from the intrinsic firing period *T* ≈ 376 *ms*. Assuming *ISI = T* seems reasonable given that inhibition acting on the silent cell decays exponentially on a much shorter timescale *τ_κ_* than the duration of the ISI. Therefore, once the silent cell is released its trajectory quickly approaches the spiking limit cycle. Naturally the above assumption requires a sufficiently small *τ_κ_*, and fails when *τ_κ_* is large. In the Supplementary Material 3 we numerically explore how different values of *τ_κ_* affect the *ISIs* of the active cell. Finally, assuming *ISI* = *T* allows us to ignore the non-linear membrane dynamics during the active phase, and to construct the evolution of the synaptic variables iteratively from spike to spike.

**Figure 4:**
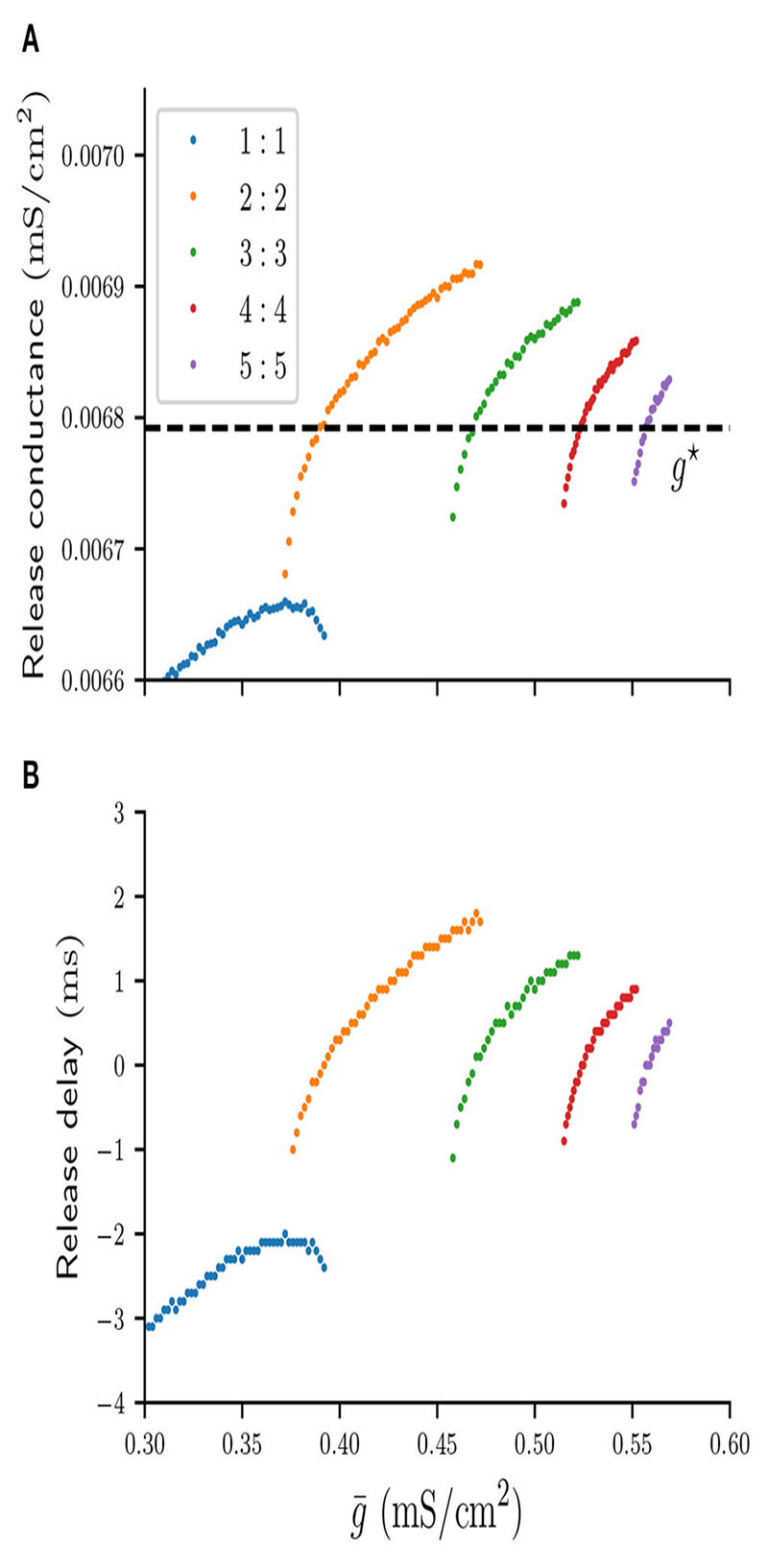
Numerically computed values of the release conductance **(A)** and release delay **(B)** for various *n* : *n* solutions and values $\bar{g}.$ The dashed line indicates the analytical approximation of the release conductance by *g*^★^.

Our second assumption states that the silent cell is released and spikes as soon as the total inhibitory conductance $\bar{g}s$ acting on it drops below some threshold value. We call this critical threshold value the “release conductance,” and define it as the value of $\bar{g}s$ at the time when the voltage of the silent cell first crosses the firing threshold *v_θ_*, that is when that cell is released and fires its first spike. Recall that when a cell is silent its *v*- and w-nullclines intersect at a stable fixed point and $\bar{g}s>g_{\text{bif}\;}.$ A sufficient condition for the silent cell to be released is therefore $\bar{g}s>g_{\text{bif}\;}.$ However, depending on the topology of the stable manifold, the (*v, w*)-trajectory of the silent cell can escape the stable fixed point and allow the cell to produce a spike for $\bar{g}s>g_{\text{bif}\;}.$ In this case the value of the release conductance depends on the type of *n* : *n* solution and the coupling strength $\bar{g}.$ For any stable *n* : *n* solution in Figure 3 we can compute an associated release conductance numerically by recording the value of $\bar{g}s$ at the time of the first spike of the silent cell. Such values of the release conductance are shown in Figure 4A, and the graph suggests that as *n* increases, the value of the release conductance converges to some constant conductance value *g*^★^ ≈ 0.0068 *mS/cm*^2^. Here *g*^★^ is the value of $\bar{g}s$ at the end of a cycle of a suppressed solution, just before the active cell spikes. Using *g*^★^ as a constant approximation for the release conductance will allow us to formulate a scalar condition that predicts the release time of the silent cell. Moreover, using *g*^★^ is convenient because its exact value can be derived explicitly, as will be shown in the following section.

Assuming a constant release conductance for all *n* : *n* solutions will naturally introduce some error in the prediction of the release time of the silent cell. We can compute that error for any associated solution in Figure 4A by calculating the time interval between the first spike of the silent cell and the time when $\bar{g}s$ first crosses *g*^★^. We will call this time interval the “release delay.” Figure 4B shows the numerically computed graph of such release delays. For *n* > 1 the absolute delays are smaller than 2 *ms*. Therefore, using

(7) $$\bar{g}s=g^{★}$$

as a constant release condition for all *n : n* solutions allows us to accurately predict the timing of the release of the silent cell. And to simplify the terminology, from now on we will refer to Equation (7) simply as the “release condition.”

In summary: We assume that the release condition is sufficient to predict when the silent cell is released. Due to the symmetry of *n* : *n* solutions the release occurs at exactly half the period of the full cycle. The release time therefore uniquely determines the type of *n* : *n* solution. Furthermore, computation of the release time does not depend on the membrane nor the synaptic dynamics of the silent cell. Instead, the solution of the synaptic variable *s* of the active cell is sufficient to predict when $\bar{g}s=g^{★}$ is satisfied. Finally, the value of s at each spike time is determined by the evolution of the depression variable *d* of the active cell. Constructing a solution of *d* during the active phase of either cell will therefore uniquely determine the solution of the full eight-dimensional network. However, finding the solution *d* requires us to know the initial value *d*(0) at the start of a cycle at *t* = 0. In the next section we will construct a scalar return map that tracks these initial values *d*(0) from cycle to cycle of stable *n* : *n* solutions.

### 3.3 Construction of the Scalar Poincaré Map

**Figure 5:**
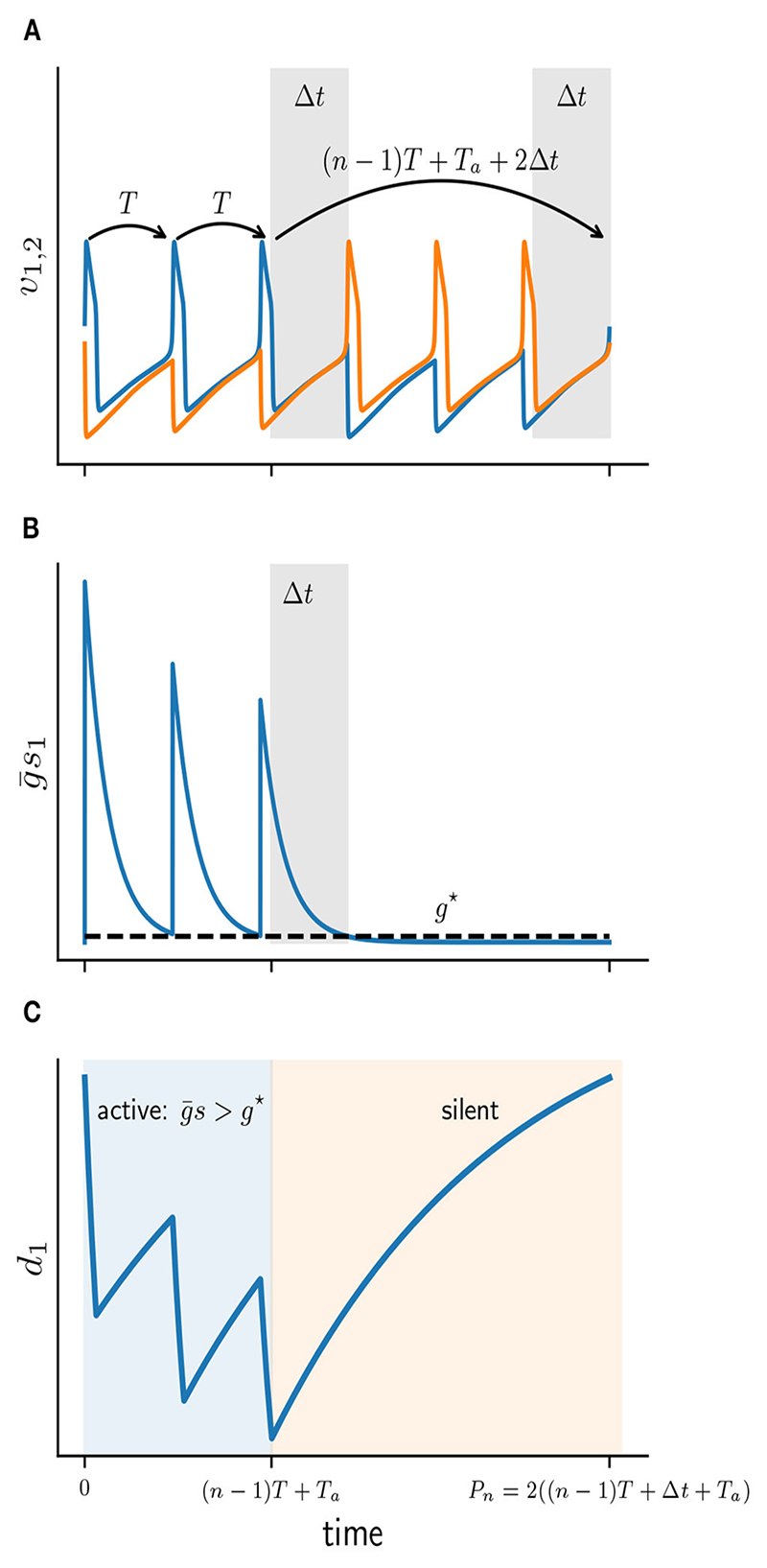
Schematic diagram of the active and silent phases for a 3 : 3 solution. **(A)** Membrane potentials of cell 1 (*v*_1_ blue) and cell 2 (*v*_2_ orange). Gray patches depict Δ*t* intervals. **(B)** Total synaptic conductance of cell 1 $\left(\bar{g}s_{1}\right)$ as it crosses the release conductance *g*^★^. **(C)** Solution *d*_1_(*t*) of depression variable of cell 1, during active (blue) and silent phases (orange).

In this section we construct the scalar Poincaré map Π*_n_* : *d*^★^ ↦ *d*^★^. Here the discrete variable *d*^★^ tracks the values of the continuous depression variable *d* at the beginning of each *n* : *n* burst. The map Π*_n_* therefore describes the evolution of *d*, of either of the two cells, from the beginning of one cycle to the beginning of the next cycle. To simplify the map construction we will assume that an active cell fires exactly *n* times before it becomes silent. We will construct Π*_n_* by evolving *d* first during the active phase and then during the silent phase of the *n* : *n* limit cycle. The terms “active” and “silent” phases will be defined in terms of the state of the depression variable. During the active phase the depression variable of the active cell both decays and recovers, while during the silent phase it only recovers. First, let us give explicit definitions of the active and silent phases of a burst. A schematic illustration of both phases is given in Figure 5.

Suppose that at *t* = 0 cell 1 becomes active with some initial *d*(0). Cell 1 then fires *n* spikes at the uncoupled period *T* = *T_a_* + *T_s_*. Let *s(t)* and *d(t)* be the corresponding solutions of the synaptic and depression variables of cell 1. After *n* spikes the total conductance $\bar{g}s(t)$ acting on the silent cell 2 has decayed sufficiently to satisfy the release condition (Equation 7). That is at some time *t* = (*n* – 1)*T* + *T_a_* + Δ*t*, where Δ*t* < *T_s_* will be determined below, we have $\bar{g}s(t)=g^{★}$ [6]. Cell 2 is then released and prevents cell 1 from further spiking. Once released, cell 2 also fires *n* spikes until cell 1 becomes active once again. Let *P_n_* denote the full cycle period of a *n* : *n* solution:

(8) $$P_{n}=2[(n-1)T+T_{a}+\text{Δ}t].$$

We can now define the active and silent phases of cell 1 explicitly. The active phase of a burst is the interval that lasts from the first spike time up until the beginning of the silent phase of the last spike, that is for time 0 < *t* < (*n* – 1)*T* + *T_a_*. During the active phase of cell 1, the silent cell 2 is inhibited sufficiently strong to prevent it from firing, hence $\bar{g}s=g^{★}.$ The silent phase of cell 1 is the remaining duration of the cycle when the cell is not firing, that is for (*n* – 1)*T* + *T_a_* < *t* < *P_n_*. The silent phase lasts for (*n* – 1)*T* + *T_a_* + 2Δ*t*.

Note that only the silent phase depends on Δ*t*, which will play a central role in the construction of Π*_n_*. From Equation (8) Δ*t* can be computed as

(9) $$\text{Δ}t=\frac{1}{2}P_{n}-(n-1)T-T_{a}.$$

**Figure 6:**
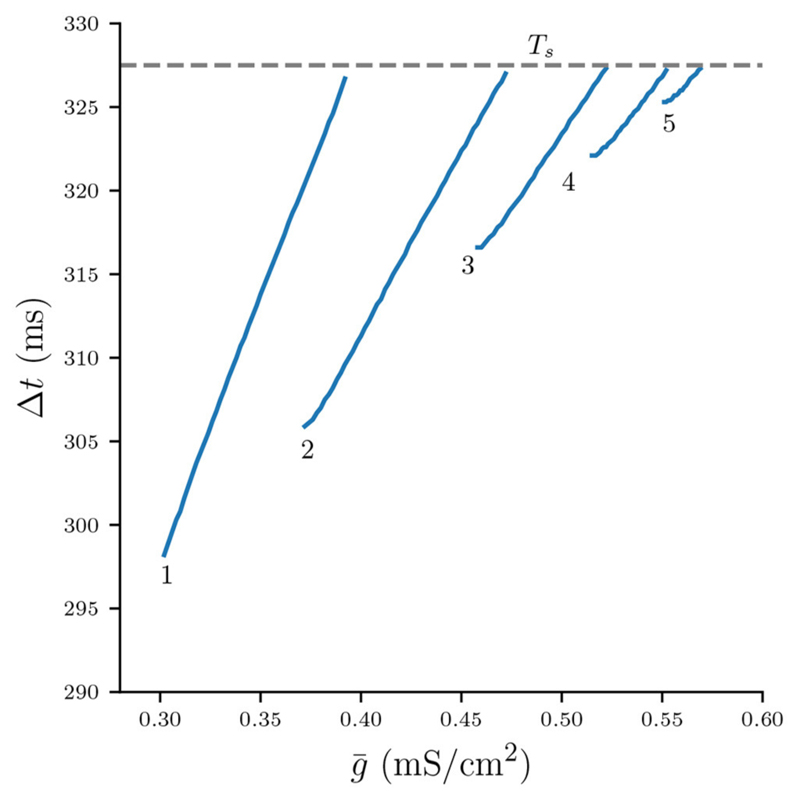
Numerically computed bifurcation diagram of Δ*t* for varying $\bar{g}.$ Each continuous branch is associated with a stable *n* : *n* burst solution. Increasing $\bar{g}$ increases At until the solutions bifurcate at Δ*t* ≈ *T_s_*.

We can use Equation (9) and the numerically computed bifurcation diagram of the period for stable *n* : *n* solutions in Figure 3 to obtain the graph of Δ*t* as a function of $\bar{g}$ (Figure 6). Each continuous branch of Δ*t* is monotonically increasing and corresponds to a *n* : *n* burst: Stronger coupling $\bar{g}$ increases the total synaptic conductance $\bar{g}s$ that acts on the silent cell, thus delaying its release. It is easy to see that for any *n*-branch we have Δ*t* < *T_s_*: Once Δ*t* crosses *T_s_*, the active cell can “squeeze in” an additional spike and the solutions bifurcate into a (*n*+1) : (*n*+1) burst.

Distinguishing between the active and silent phases of a *n* : *n* cycle allows us to describe the dynamics of the depression variable *d* explicitly for each phase. As can be seen from Figure 5C, during the active phase *d* depresses when *v* > *v_θ_* and recovers when *v* < *v_θ_*. In contrast, during the silent phase *d* only recovers and does not depress. Given the initial *d*^★^ = *d*(0) at the beginning of the cycle and the number of spikes in the active phase *n*, we can now construct the burst map Π*_n_*. The map

(10) $${\text{Π}}_{n}(d^{★})=Q_{n}[F_{n}(d^{★})]$$

is a composition of two maps. Map

(11) $$F_{n}:d^{★}\mapsto \text{Δ}t$$

models the evolution of *d* in the active phase. *F_n_* takes an initial value *d*^★^ and calculates Δ*t*. Map

(12) $$Q_{n}:\text{Δ}t\mapsto d^{★}$$

models the recovery of *d* in the silent phase. Given some Δ*t* map *Q_n_* computes *d*^★^ at the start of the next cycle.

Our aim in the following analysis is to elucidate the properties of Π*_n_* and to understand the structure of its parameter space by exploring how the stable and unstable fixed points of Π*_n_* are created. To that effect it will be useful to include not only positive, but also negative values of *d*^★^ to the domain of Π_n_. But it is important to add that values *d*^★^ < 0 are biologically impossible as the depression variable models a finite pool of neurotransmitters, and therefore must be positive. Because Π*_n_* maps first from *d*^★^ to Δ*t*, and then back to *d*^★^, we will also consider negative values of Δ*t*, interpreting them as *n* : *n* solutions with partially overlapping bursts. As will become evident, Δ*t* < 0 is only a formal violation of the biological realism of the map Π_n_, as numerically stable *n* : *n* solutions of the full system of ODEs only exist for Δ*t* > 0.

We start the construction of Π*_n_* by first considering the active phase and building the map *F_n_*. At each spike time *t_k_* where *d*(*t_k_*) = *d_k_*, variable *d* decays first for the duration of *T_a_*, as described by the solution to Equation (5). At *t* = *t_k_* + *T_a_* we have

(13) $$d(t_{k}+T_{a})=d_{k}e^{-T_{a}/\tau_{b}}.$$

The depression variable then recovers for *T_s_* until *t*_*k*+1_, where for 0 < *t* < *T_s_*:

(14) $$d(t_{k+1})=1-(1-d_{k}e^{-T_{a}/\tau_{b}})e^{-t/\tau_{a}}.$$

By substituting *t* = *T_s_* we can build a linear map that models the depression of *d* from spike time *t_k_* to the subsequent spike time *t*_*k*+1_ during the active phase:

(15) $$d_{k+1}=\lambda \rho d_{k}+(1-\rho )\text{,}$$

where to keep the notation simple we let

(16) $$\lambda :=\exp(-T_{a}/\tau_{b}),$$

(17) $$\rho :=\exp(-T_{s}/\tau_{a}).$$

Given constant *T_a_* and *T_s_*, the derived parameter *λ* determines how much the synapses depresses when *v* > *v_θ_*, while *ρ* determines how much it recovers when *v* < *v_θ_*. Since 0 < *λ*, *ρ* < 1, the map in Equation (15) is increasing and contracting, with a fixed point at

(18) $$d_{s}=\frac{1-\rho }{1-\lambda \rho },$$

where 0 < *d_s_* < 1. The value *d_s_* is the maximum depression value that can be observed in the suppressed solution where the active cell fires at its uncoupled period *T* (see Figure 2E). Using the release condition in Equation (7) allows us to derive the value of the minimum coupling strength that will produce the full suppressed solution, denoted as ${\bar{g}}_{s}.$ Solving Equation (7) for *s(t)* with *t* = *T_s_* and setting the initial value *s*(0) = *d_s_λ* then gives us the aforementioned approximation of the release conductance *g*^★^:

(19) $${\bar{g}}_{s}d_{s}\lambda e^{-T_{s}/\tau_{\kappa }}=g^{★}\approx 0.0068mS/cm^{2}.$$

By substituting the definition of *d_s_* in Equation (18) and rearranging, we can also write ${\bar{g}}_{s}$ as a function of *λ* and *ρ* :

(20) $${\bar{g}}_{s}(\lambda ,\rho )=\frac{1/\lambda -\rho }{1-\rho }e^{T_{s}/\tau_{\kappa }}g^{★}.$$

Note that the above dependence of ${\bar{g}}_{s}$ on *λ* is linear and monotonically decreasing. Increasing *λ* reduces the strength of the depression of the active cell. This in turn allows the active cell to fully suppress the silent cell at smaller values of $\bar{g}.$

Solving Equation (15) gives us the linear map *δ_n_*, that for some initial *d*^★^ computes the depression at the *n*th spike time, that is *d(t_n_)*:

(21) $$\delta_{n}(d^{★})={\left(\lambda \rho \right)}^{n-1}d^{★}+(1-\rho )\sum_{i=0}^{n-2}{\left(\lambda \rho \right)}^{i}.$$

Since *λ* < 1, function *δ_n_* is a linearly increasing function of *d*^★^ with a fixed point at *d_s_* for all *n*. Having identified *d* after *n* spikes, we can now use the release condition $\bar{g}s=g^{★}$ (Equation 7) to find Δ*t*. At the last (*n*th) spike of the active phase at time *t_n_* = (*n* – 1)*T* the synapse variable *s* is set to the respective value of *d*(*t_n_*) = *δ_n_*(*d*^★^), and mirrors the value of *d* for the duration of *T_a_*. At the end of the active phase at time *t_n_* + *T_a_* variable *d* has decayed to *δ_n_*(*d*^★^)*λ*, therefore

(22) $$s(t_{n}+T_{a})=\delta_{n}(d^{★})\lambda .$$

Finally *s* decays exponentially for Δ*t* < *T_s_*. Solving (Equation 6) with initial condition *s*(0) = *δ_n_*(*d^★^*)*λ* yields:

(23) $$s(\text{Δ}t)=\delta_{n}(d^{★})\lambda e^{-\text{Δ}t/\tau_{\kappa }}.$$

Substituting *s*(Δ*t*) into s of the release condition (Equation 7) gives then

(24) $$\bar{g}\delta_{n}(d^{★})\lambda e^{-\text{Δ}t/\tau_{\kappa }}=g^{★}.$$

Our assumption of the release condition guarantees that the silent cell 2 spikes and becomes active when $\bar{g}s—g^{★}$ crosses zero. Solving (Equation 24) for Δ*t* allows us to compute Δ*t* as a function of *d^★^*, which defines the map *F_n_*:

(25) $$F_{n}(d^{★}):=\tau_{\kappa }\ln(\frac{\bar{g}}{g^{★}}\lambda \delta_{n}(d^{★}))=\text{Δ}t.$$

**Figure 7:**
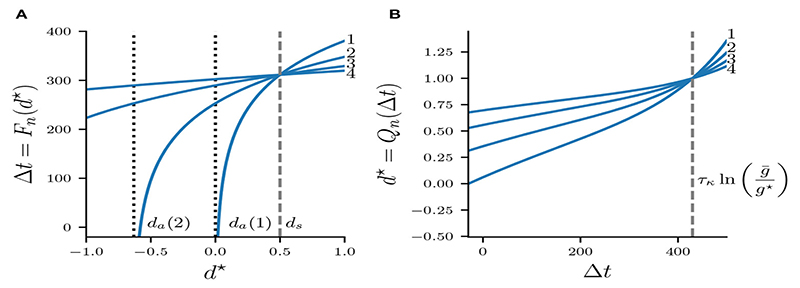
Maps *F_n_* (**A**) and *Q_n_* **(B)** for $\bar{g}=0.5\text{mS}/{\text{cm}}^{2}$ and *n* = 1,2,3,4. Curves *F_n_* intersect at *d*^★^ = *d_s_* which is indicated by a dashed vertical line. Curves *Q_n_* intersect at $\text{Δ}t=\tau_{\kappa }\ln(\bar{g}/g^{★}).$

Figure 7A shows *F_n_* for various *n*, which is a strict monotonically increasing function of *d^★^* as well as $\bar{g}.$ Larger values of *d^★^* and $\bar{g},$ respectively, cause stronger inhibition of the silent cell, and therefore prolong its release time and the associated Δ*t*. Map *F_n_* is defined on *d^★^* > *d_a_*, where *d_a_* is a vertical asymptote found by solving *δ_n_*(*d^★^*) = 0 in Equation (21) for *d^★^*, which yields

(26) $$d_{a}(n)=-\frac{(1-\rho )\sum_{i=0}^{n-2}{\left(\lambda \rho \right)}^{i}}{{\left(\lambda \rho \right)}^{n-1}}\leq 0.$$

We now turn to the construction of map *Q_n_*, which describes the recovery of the depression variable during the silent phase. As we have identified earlier, the recovery of *d* in the silent phase of a *n* : *n* solution starts at time *t_n_* + *T_a_* and lasts for the duration of (*n* – 1)*T* + *T_a_* + 2Δ*t*. Substituting that duration into the solution of *d* (Equation 5) with the initial condition *d*(0) = *δ_n_*(*d^★^*)*λ* yields the map *Q_n_*:

(27) $$Q_{n}(\text{Δ}t):=1-[1-\delta_{n}(d^{★})\lambda ]e^{-[(n-1)T+T_{a}+2\text{Δ}t]/\tau_{a}}.$$

We can find *δ_n_*(*d^★^*), i.e., the value of *d* at the nth spike time, by rearranging the release condition in Equation (24):

(28) $$\delta_{n}(d^{★})=\frac{1}{\bar{g}\lambda }g^{★}e^{\text{Δ}t/\tau_{\kappa }}.$$

Map *Q_n_* is shown in Figure 7B for various values *n*. Note that *Q_n_* is monotonically increasing as larger values Δ*t* imply a longer recovery time, and hence *Q_n_* grows without bound. All curves *Q_n_* intersect at some $\text{Δ}t=\tau_{\kappa }\ln[\bar{g}/g^{★}]$ where

(29) $$Q_{n}[\tau_{\kappa }\ln(\frac{\bar{g}}{g^{★}})]=1.$$

As we will show in the next section, all fixed points of the full map Π_n_ occur for *d^★^* < 1. We will therefore restrict the domain of *Q_n_* to $\left(-\infty ,\tau_{\kappa }\ln[\bar{g}/(g^{★})]\right)$ and the codomain to (–∞, 1). Additionally, while values Δ*t* > *T* will be helpful in exploring the geometry of Π*_n_*, recall from Figure 6 that in the flow system *n* : *n* solutions bifurcate into (*n* + 1) : (*n* + 1) solutions exactly when Δ*t* = *T_s_*, and we will address this concern in the last part of our map analysis.

**Figure 8:**
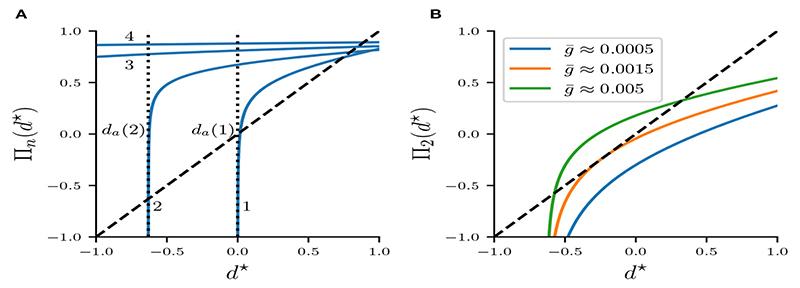
Map Π*_n_* : *d*^★^ → *d*^★^. **(A)** Π*_n_* for *n* = 1,2,3,4 at $\bar{g}=0.5\text{mS}/{\text{cm}}^{2}.$ **(B)** Π_2_ with *n* = 2 for various $\bar{g}.$ The identity function is illustrated by a diagonal line.

Having found *F_n_* and *Q_n_*, we can now construct the full map Π*n*(*d^★^*) = *Q_n_*[*F_n_*(*d^★^*)]:

(30) $$\prod_{H}(d^{★})=1-[1-\delta_{n}(d^{★})\lambda ]{\left[\frac{\bar{g}}{g^{★}}\delta_{n}(d^{★})\lambda \right]}^{-\tau }e^{-[(n-1)T+T_{a}]/\tau_{a}},$$

where we substituted *τ* = 2*τ_k_*/*τ_a_*. Recall that *δ_n_*(*d^★^*) and *g^★^* are obtained from Equations (21) and (19), respectively. Since *d* is the slowest variable of the system and *τ_a_* ≫ *τ_κ_*, we will also assume *τ* < 1. Figure 8A depicts Π*_n_* for various *n*. Intersections of Π*_n_* with the diagonal are fixed points of the map. Figure 8B shows Π_2_ with *n* = 2. Varying the synaptic strength $\bar{g}$ moves the curves Π*_n_* up and down the (*d^★^*, Π*_n_*)-plane. For $\bar{g}<0.0015mS/cm^{2}$ map Π_2_ has no fixed points. As $\bar{g}$ is increased to $\bar{g}\approx 0.0015mS/cm^{2},$ curve Π_2_ coalesces with the diagonal tangentially. When $\bar{g}>0.0015\;mS/cm^{2},$ a pair of fixed points emerge, one stable and one unstable fixed point, indicating the occurrence of a fold bifurcation of maps.

Π*_n_* is monotonically increasing with respect to $\bar{g}$ and also *d*^★^:

(31) $$\frac{d{\text{Π}}_{n}}{d\bar{g}}>0,$$

(32) $$\frac{d{\text{Π}}_{n}}{dd^{★}}>0,$$

The monotonicity of Π*_n_* w.r.t. $\bar{g}$ is evident from Equation (30), while the monotonicity w.r.t. $\bar{g}$ follows from the monotonicity of both *Q_n_* and *F_n_*. In the following sections we will heavily rely on this monotonicity property of Π*_n_*. Just as *F_n_*, curves Π*_n_* spawn at the asymptote da (Equation 26), and because

(33) $$\underset{\bar{g}\to \infty }{\lim}{\text{Π}}_{n}=1\;\text{for all}\;\;n\text{,}$$

fixed points of Π*_n_* lie in *d_a_*, 1).

### 3.4 Existence and Stability of Fixed Points

We introduce the fixed point notation $d_{f}^{★}$ with ${\text{Π}}_{n}(d_{f}^{★})=d_{f}^{★}.$ The existence of fixed points $d_{f}^{★}$ for $\bar{g}$ sufficiently large can be shown from the strict monotonicity of Π*_n_* with respect to $\bar{g}$ and *d^★^* (Equations 32, 31), as well as the fact that the slope of Π*_n_* is monotonically decreasing,

(34) $${\left(\frac{\text{d}}{\text{d}d^{★}}\right)}^{2}{\text{Π}}_{n}<0\text{.}$$

In the limit *d^★^* → *d_a_* the value of Π*_n_* decreases without bound for any $\bar{g}>0.$ In the limit $\bar{g}\to 0,{\text{Π}}_{n}$ also decreases without bound, but as $\bar{g}\to \infty$ values of Π*_n_* approach 1. It follows from Equation (31) and the intermediate value theorem that for some $\bar{g}$ large enough Π*_n_* intersects the diagonal. Moreover, because Π*_n_* and its slope are monotonic with respect to *d^★^*, there exists some critical fixed point $\left(d_{b}^{★},{\bar{g}}_{b}\right)$ where Π*_n_* aligns with the diagonal tangentially with

(35) $${\text{Π}}_{n}(\;d_{b}^{★},{\bar{g}}_{b})=d_{b}^{★},$$

(36) $$\frac{d}{dd^{★}}{\text{Π}}_{n}(d^{★}{\;}_{b},{\bar{g}}_{b})=1.$$

### 3.5 Fold Bifurcations of Maps

Fixed points of Π*_n_* satisfy the fixed point equation

(37) $${\text{Φ}}_{n}(d^{★},\bar{g}):={\text{Π}}_{n}(d^{★},\bar{g})-d^{★}=0.$$

**Figure 9:**
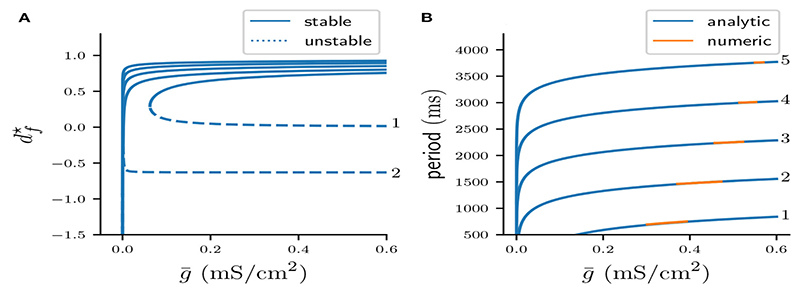
**(A)** Fold bifurcation diagrams of stable (continuous curves) and unstable (dotted curves) fixed points of Π*_n_* for varying *n*. **(B)** Cycle periods computed from stable fixed points of Π*_n_* (blue), and the corresponding periods from stable *n* : *n* solutions acquired via numerical integration of the system of ODEs (orange).

As we have already shown, for $\bar{g}>{\bar{g}}_{b}(n)$ solutions to Equation (37) exist in pairs of stable and unstable fixed points. Solving (Equation 37) explicitly for *d^★^* is not trivial, but solving for $\bar{g}$ is straightforward and given by $\bar{g}=G_{n}(d^{★}),$ where

(38) $$G_{n}(d^{★}):=\frac{g^{★}}{\delta_{n}(d^{★})\lambda }(\frac{\left[1-\lambda \delta_{n}(d^{★})\right]}{1-d^{★}}e^{-[(n-1)T+T_{a}]/\tau_{a}}{)}^{1/\tau }$$

is defined for *d^★^* < 1 and *δ_n_*(*d^★^*) > 0. Plotting *d^★^* against $\bar{g}$ gives the fixed point curves, which are shown in Figure 9A. Note the typical quadratic shape of a fold bifurcation of maps. It is also evident that the fold bifurcations occur for increasingly smaller $\bar{g}$ as *n* is increased. Moreover, the graph suggests that for *n* > 1 unstable fixed points have negative values of *d^★^*.

Equation (38) also allows us to find the critical fixed point connected with the fold bifurcation, namely $[d_{b}^{★}(n),{\bar{g}}_{b}(n)],$ which is the global minimum of $G_{n}(d_{f}^{★}):$

(39) db★(n)=argmin Gn(df★),

(40) $${\bar{g}}_{b}(n)=\min G_{n}(d_{f}^{★}).$$

Function *G_n_* is strictly monotonic on the respective intervals of $d_{f}^{★}$ that correspond to the stable and unstable fixed points, that is

(41) $$\frac{dG_{n}}{dd_{f}^{★}}>0,\;\;\text{for}\;d_{f}^{★}>d_{b}^{★}(n)\;\text{stable}\;,$$

(42) $$\frac{dG_{n}}{dd_{f}^{★}}<0,\;\;\text{for}\;d_{f}^{★}<d_{b}^{★}(n)\;\text{unstable,}\;$$

which allows us to express the stable and unstable fixed points as the inverse of *G_n_* on their respective intervals of $d_{f}^{★}.$ Because we are primarily interested in the stable fixed points $d_{f}^{★}>d_{b}^{★}(n).$ we define the stable fixed point function $d_{f}^{★}=\phi_{n}(\bar{g})$ as

(43) $$\phi_{n}(\bar{g}):=G_{n}^{-1}(\bar{g}).$$

Function $\phi_{n}(\bar{g})$ is also monotonic, and is therefore straightforward to compute numerically. We use the Python package Pynverse [21] for that purpose.

Having found the stable fixed points $d_{f}^{★}$ as a function of $\bar{g},$ we can now compute the associated cycle period. Recall that the period is given by Equation (8), which can be written as a function of $\bar{g}:$

(44) $$P_{n}(\bar{g})=2((n-1)T+T_{a}+F_{n}\underset{d_{f}^{★}}{\underset{︸}{\phi_{n}(\bar{g}),\bar{g}}}),$$

where map *F_n_* (Equation 25) calculates Δ*t* given a stable fixed point $d_{f}^{★}=\phi_{n}(\bar{g}),$ Figure 9B shows the period $P_{n}(\bar{g})$ computed from Equation (44) versus the cycle period of stable *n* : *n* solutions, computed from numerically integrating the full system of ODEs. The overlap between blue and orange curves suggests that stable fixed points of Π*_n_* accurately predict the cycle period of stable solutions of the flow system.

It is evident from Figure 9A that *ϕ_n_* is strictly increasing with $\bar{g}.$ This property follows directly from the quadratic normal form of the fold bifurcation, but can also be shown using implicit differentiation and the fixed point equation ${\text{Φ}}_{n}[\phi_{n}(\bar{g}),\bar{g}]=0$ in Equation (37). For $d_{f}^{★}=\phi_{n}(\bar{g})>d_{b}(n)$ we get:

(45) $$\frac{d\phi_{n}}{d\bar{g}}=-\frac{\partial {\text{Φ}}_{n}/\partial \bar{g}}{\partial {\text{Φ}}_{n}/\partial d^{★}}=\frac{\partial {\text{Π}}_{n}/\partial \bar{g}}{1-\partial {\text{Π}}_{n}/\partial d^{★}}>0.$$

The inequality follows from $\partial {\text{Π}}_{n}/\partial \bar{g}>0$ and the fact that *∂Π_n_/∂* *d^★^* < 1 for *d^★^* > *d_b_(n)*. Equation (45) allows us to explain why the period *P_n_* increases with $\bar{g},$ as seen in Figure 9B. Differentiating *P_n_* gives:

(46) $$\frac{dP_{n}}{d\bar{g}}=2\nabla F_{n}(d_{f}^{★},\bar{g})\cdot [\begin{array}{c} \partial \phi_{n}/\partial \bar{g}\\ 1 \end{array}]>0,$$

where the partial derivatives of $F_{n}(d_{f}^{★},\bar{g})$ are:

(47) $$\frac{\partial F_{n}}{\partial d_{f}^{★}}=\tau_{\kappa }\frac{{\left(\lambda \rho \right)}^{n-1}}{\delta_{n}(d_{f}^{★})}>0.$$

(48) $$\frac{\partial F_{n}}{\partial \bar{g}}=\frac{\tau_{\kappa }}{\bar{g}}>0.$$

Equation (46) and (47) have an intuitive biological interpretation: Increasing the coupling strength between the neurons leads to overall stronger inhibition of the silent cell, which delays its release and leads to a longer cycle period. The latter allows more time for the synapse to depress in the active phase and recover in the silent phase, resulting in overall larger values of $d_{f}^{★},$ that is weaker depression at the burst onset.

While fixed points of our Poincaré map predict the cycle period of the flow system excellently, its construction relies on the strong assumption that the active phase contains exactly *n* spikes. As is evident from Figure 9B this assumption is clearly violated in the flow system, as stable *n* : *n* bursts exists only on certain parameter intervals of $\bar{g}.$ The multi-stability of fixed points of maps Π*_n_* in Figure 9B does therefore not imply a similar multi-stability of the flow system. In the last sub-section we will analyze the mechanisms that guide how the stable *n* : *n* are created and destroyed, and use our previous analysis to derive the corresponding parameter intervals of $\bar{g}$ where such solutions exist.

### 3.6 Stable Solution Branch Borders

Let ${\bar{g}}_{L}(n)$ and ${\bar{g}}_{R}(n)$ denote the left and right parameter borders on $\bar{g}$ where stable *n* : *n* solutions exist. That is, as $\bar{g}$ is increased stable *n* : *n* solutions are created at ${\bar{g}}_{L}(n)$ and destroyed at ${\bar{g}}_{R}(n).$ When $\bar{g}$ is reduced beyond ${\bar{g}}_{L}(n),$ *n* : *n* solutions bifurcate into (*n* – 1) : (*n* – 1) solutions, while when $\bar{g}$ is increased beyond ${\bar{g}}_{R}(n),$ *n* : *n* solutions bifurcate into (*n* + 1) : (*n* + 1) solutions. Let us briefly recap our observations regarding ${\bar{g}}_{L}(n)$ and ${\bar{g}}_{R}(n)$ from the numerical bifurcation diagram in Figure 9B. For *n* > 1 there are the following relations:

(49) $${\bar{g}}_{L}(n)<{\bar{g}}_{R}(n),$$

(50) $${\bar{g}}_{L}(n)<{\bar{g}}_{R}(n+1)\;\text{and}\;{\bar{g}}_{R}(n)<{\bar{g}}_{L}(n+1)\text{,}\;$$

(51) $${\bar{g}}_{L}(n)<{\bar{g}}_{R}(n)$$

(52) $${\bar{g}}_{L}(n)<{\bar{g}}_{L}(n+1)\;\text{and}\;{\bar{g}}_{R}(n)<{\bar{g}}_{R}(n+1)$$

Equations (49, 50) are self-explanatory. Equation (51) formally describes occurrence of co-existence between stable *n* : *n* and (*n* + 1) : (*n* + 1) solutions. Equation (52) implies that the parameter interval on $\bar{g}$ of *n* : *n* solutions decreases with *n*, in other words, bursts with more spikes occur on increasingly smaller intervals of the coupling strength. All of the above relations are reminiscent of the bifurcation scenario of type period increment with coexistent attractors, first described for piecewise-linear scalar maps with a single discontinuity by Avrutin and colleagues [e.g., see 22–24]. While our maps Π*_n_* are fully continuous, the above observation suggests that a different piecewise-linear scalar map that captures such period increment dynamics of the full system might exist. We will explore what such a map might look like in the discussion.

Let us now find algebraic equations that will allow us to calculate the critical parameters ${\bar{g}}_{L}(n)$ and ${\bar{g}}_{R}(n)$ associated with the left and right *n* : *n* branch borders. Recall that the period *P_n_* derived from the fixed points of Π*_n_* is an increasing function of $\bar{g}$ (Equation 46). That is, as the coupling strength increases, it takes longer for the total synaptic conductance to fall below the value of the release conductance, which delays the release of the silent cell, and Δ*t* becomes larger. When Δ*t* > *T_s_*, the active cell can produce another spike and the solution bifurcates into a (*n* + 1) : (*n* + 1) solution. Note, however, that at ${\bar{g}}_{L}(n)$ the bifurcation into a (*n* – 1) : (*n* – 1) does not occur at Δ*t* = 0. Here the mechanism is different: A sufficient reduction of $\bar{g}$ causes the total synaptic conductance to drop below the release conductance in the *previous ISI*, which allows the silent cell to be released one spike earlier.

Using the above reasoning we can now formulate the conditions for both bifurcations at ${\bar{g}}_{L}(n)$ and ${\bar{g}}_{R}(n).$ As in the previous sections, we will only restrict ourselves to the analysis of the stable fixed points given implicitly by $d_{f}^{★}=\phi_{n}(\bar{g})$ (Equation 43). At the right bifurcation border ${\bar{g}}_{R}(n)$ we have Δ*t* = *T_s_*, and after substituting our *F_n_* map (Equation 25) this translates into

(53) $${\bar{g}}_{L}(n+1)<{\bar{g}}_{R}(n)$$

which lets us define a function

(54) $$\;{\bar{g}}_{R}(n+1)-{\bar{g}}_{L}(n+1)<{\bar{g}}_{R}(n)-{\bar{g}}_{L}(n)$$

whose root is the desired right bifurcation border ${\bar{g}}_{R}(n).$ In case of the left bifurcation border at ${\bar{g}}_{L}(n),$ the release condition is satisfied just before the active cell has produced its *n*th spike, where total synaptic conductance is given by

(55) $$\bar{g}\delta_{n-1}[\phi_{n}(\bar{g})]\lambda e^{-T_{s}/\tau_{\kappa }}=g^{★},$$

which can be rewritten as a function

(56) $$L_{n}(\bar{g}):=\bar{g}\delta_{n-1}[\phi_{n}(\bar{g})]\lambda e^{-T_{s}/\tau_{\kappa }}-g^{★},$$

whose root is ${\bar{g}}_{L}(n).$ Both $R_{n}\;\text{and}\;L_{n}$ are increasing with respect to $\bar{g},$ which makes finding their roots numerically straightforward.

**Figure 10:**
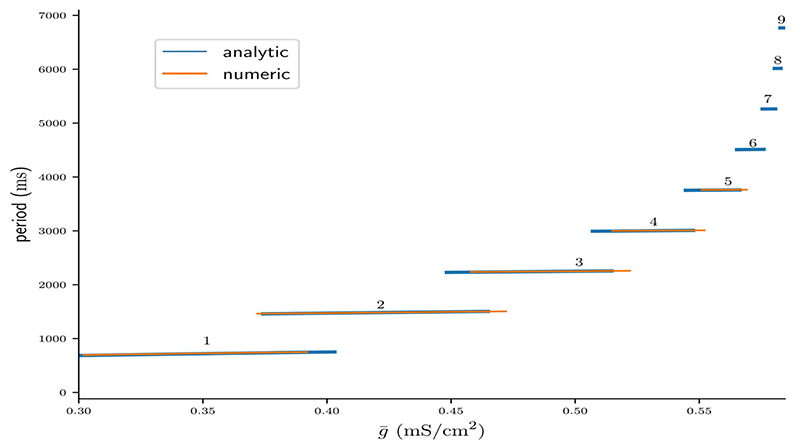
Bifurcation diagram of the period of stable *n* : *n* solutions computed analytically from fixed points of Π*_n_*, plotted on the respective intervals of $\bar{g}\in [{\bar{g}}_{L}(n),{\bar{g}}_{R}(n)]$ (blue), and computed from numerical integrations of the ODEs (orange).

Figure 10 shows the period $P_{n}(\bar{g})$ as predicted by the fixed points of Π*_n_* (Equation 44) plotted on their respective intervals $\bar{g}\in [{\bar{g}}_{L}(n),{\bar{g}}_{R}(n)]$ (blue), as well as the cycle period acquired from numerical integration of the full system of ODEs (orange).

Here ${\bar{g}}_{L}(n)$ and ${\bar{g}}_{R}(n)$ were computed from Equations (56) and (54), respectively. Note that the width of *n* : *n* branches decreases with *n*, which confirms the inequality in Equation (52). That is, bursts with more spikes occur on increasingly smaller intervals of g, which can be interpreted as a lost of robustness with respect to the coupling strength of long-cyclic solutions. We also note the occurrence of bistability between pairs of *n* : *n* and (*n* + 1) : (*n* + 1) branches, which also confirms our initial observation in Equation (51). As previously observed in Figure 9B our maps prediction of the cycle period is accurate. However, the mismatch in the left and right branch borders is significant. This mismatch might be due to the millisecond release delay error (Figure 4B) induced by our assumption of a constant release conductance for all *n* : *n* solutions (see Equation 7). Another explanation for the border mismatch could be that our assumptions on the time scales of (*v, w*) vs *s*- and d-dynamics do not hold near the stability borders, and that they can only be captured by more complex approximations. Nevertheless, our map allows approximate extrapolation of the cycle period and the respective bifurcation borders where numerical integration of the ODEs would require a very small time step.

## 4 Discussion

Synaptic depression of inhibition is believed to play an important role in the generation of rhythmic activity involved in many motor rhythms such as in leech swimming [25] and leech heart beat [26], and in the lobster pyloric system [1, 2]. In inhibitory half-center CPGs, such as believed to be found in the struggling network of *Xenopus* tapdoles, synaptic depression can act as a burst termination mechanism, enabling the alternation of bursting between the two sides of the CPG [11]. Modeling can shed light on the underlying mathematical principles that enable the generation of such anti-phase bursts, and help identify the components that control this rhythm allowing it to switch between different patterns.

To study the mechanisms of burst generation in half-center CPGs we have analyzed a neuronal model network that consists of a pair of inhibitory neurons that undergo a frequency dependent synaptic depression. When the strength of synaptic inhibition between the neurons is varied, such a simple network can display a range of different *n* : *n* burst patterns. Using the timescale disparity between neuronal and synaptic dynamics, we have reduced the network model of eight ODEs to a scalar first return map Π*_n_* of the slow depression variable d. This map Π*_n_* is a composition of two maps, *F_n_* and *Q_n_*, that model the evolution of the depression during the active and silent phases of *n* : *n* solutions respectively. Both *Fn* and *Q_n_* maps are constructed by using the dynamics of a single uncoupled neuron. Fixed points of Π*_n_* are created in pairs through a fold bifurcation of maps, where the stable fixed point correspond to stable *n* : *n* burst solutions of the full two-cell system of ODEs. The results from our one-dimensional map match excellently with numerical simulation of the full network. Our results are also in line with Brown’s [7] rhythmogenesis hypothesis, namely that synaptic depression of inhibition is a mechanism by which anti-phase bursting may arise.

We have studied *n* : *n* solutions assuming that the synaptic coupling $\bar{g}$ between the two cells is symmetrical. However, Bose and Booth [6] have shown that asymmetrical coupling $\left({\bar{g}}_{1},{\bar{g}}_{2}\right)$ can result in network solutions of type *m* : *n*, where one cell fires *m* spikes, while the other *n* spikes. It is conceivable that our map construction can be extended to also capture such *m* : *n* solutions. Remember, in the case of symmetrical coupling with *n* : *n* solutions, the timecourse of the depression variables *d*_1_ and *d*_2_ were in anti-phase, and it was therefore sufficient to track only one of the two variables. To capture the full network dynamics in case of asymmetrical coupling one would also have to account for burst patterns of type *m* : *n*, where the solutions of the depression variables *d*_1_ and *d*_2_ are not simply time-shifted versions of each other. To do that, one could track the state of both variables by constructing a two-dimensional Poincaré map Π(*d*_1_, *d*_2_). While geometrical interpretation of two-dimensional maps remains challenging, there exist a number of recent studies which have employed novel geometrical analysis methods to understand the dynamics of two-dimensional maps of small neuronal networks [27–29]. Generally speaking, our map construction approach is applicable to any small network, even with more than two neurons. As long as the network dynamics occur on separable timescales the main challenges to the map construction lie in identifying the slowest variables, and finding an appropriate, simplified description of their respective timecourses. In theory, the reduction approach can be also applied to neuronal systems with more than two timescales [e.g., see 30].

In tadpoles, struggling is believed to be initiated by an increase in the firing frequency of reciprocally inhibitory commisural interneurons, which has been hypothesized to lead to stronger synaptic depression of inhibition and result in the iconic antiphase bursting [11]. It would therefore be interesting to study how varying the cell intrinsic firing period *T* could affect the network rhythm. While we have laid out the framework to perform such an investigation, due to the choice of neural model we have avoided varying *T*. Recall that *T* is a derived parameter in the Morris and Lecar [12] model, and can therefore not be varied in isolation of other model parameters. This makes verifying any analytical results from our map analysis via numerical integration of the ODEs difficult. A more abstract model such as the quadratic integrate-and-fire model [31] allows varying *T* independently of other model parameters, and could be more fitting for such an investigation.

Our simulations of the network showed that *n* : *n* solutions lose robustness as their period is increased. That is, solutions with a larger cycle period occur on increasingly smaller intervals of the coupling strength. We were able to replicate this finding by numerically finding the respective left and right borders of stable *n* : *n* branches of fixed points of Π*_n_*, and showing that the distance between these borders shrinks with *n*. We have also noted the resemblance of our bifurcation diagram to one where such *n* : *n* branches are created via the bifurcation scenario of type period-increment with co-existent attractors, first described for scalar linear maps with a discontinuity [24, 32]. It is worthwhile noting that the bifurcations of piecewise linear maps studied by Avrutin et al. [32] result from a “reinjection” mechanism [33]. Here the orbit of a map performs multiple iterations on one side of the discontinuity, before jumping to the other side and being *reinjected* back into the initial side of the discontinuity. The stark difference of such a map to our map is that reinjection allows a *single* scalar map to produce periodic solutions of varying periods. In contrast, we rely on *n* different maps Π*_n_* to describe the burst dynamics without explicitly capturing the period increment dynamics. It is therefore conceivable that despite the complexity and non-linearity of the dynamics of our two-cell network, a single piecewise-linear map might be already sufficient to capture the mechanisms that shape the parameter space of the full system. In their discussion, Bose and Booth [6] briefly outline ideas about how such a linear map could be constructed.

In addition to stable *n* : *n* solutions, the numerical continuation by Bose and Booth [6] also revealed branches of unstable *n* : *n* solutions. While we have identified fold bifurcations of our burst map, we have not found corresponding bifurcations of the flow ODE system, and have generally ignored the significance of unstable map fixed points. However, the quadratic nature of the period bifurcation curve is reminiscent of a saddle-node on an invariant circle (SNIC) bifurcation, where the oscillation period lengthens and finally becomes infinite as a limit cycle coalesces with a saddle point. SNIC bifurcations have been studied in great detail [e.g., 34], and a next step would be to provide a rigorous explanation of not only the map dynamics, but also of the flow dynamics of the ODE system.

We have shown that when the strength of the maximum synaptic conductance is varied, synaptic depression of inhibition can enable our two-cell network to produce burst solutions of different periods. This result is in line with the idea that one role of synaptic depression in the nervous system may be to allow a finite size neuronal network to participate in different tasks by producing a large number of rhythms [6, 11, 35]. To change from one rhythm to another would only require a reconfiguration of the network through changes in synaptic coupling strength. Thus short-term synaptic depression of inhibition may provide means for a network to adapt to environmental challenges without changing its topology, that is without the introduction or removal of neurons.

## Supplementary Material

Supplementary material

## Data Availability

The original contributions presented in the study are included in the article/Supplementary Material, further inquiries can be directed to the corresponding author. The source code to replicate all figures is available at https://github.com/markolenik/poincare-map-paper.
