# Supplementary material for: A Scalar Poincaré Map for Anti-phase Bursting in Coupled Inhibitory Neurons With Synaptic Depression
Source: Front Appl Math Stat. Author manuscript; Available in PMC 2024 Mar 8. (PMC7615720; doi:10.3389/fams.2022.822782)
Supplement: Supplementary material [file EMS194389-supplement-Supplementary_material.pdf]

Supplementary Material

1 MODEL EQUATIONS AND PARAMETERS

The asymptotic function for the calcium conductance m_∞ is given by

$$m_\infty(v) = \frac{1}{2}\left(1 + \tanh\left((v - v_A)/v_B\right)\right). \tag{S1}$$

The voltage and potassium nullclines, $v_\infty(v)$ and $w_\infty(v)$, are

$$v_\infty(v) = \frac{-g_l(v - v_l) - g_{ca}m_\infty(v)(v - v_{ca}) + I - \bar{g}s(v - v_s)}{g_k(v - v_k)}, \tag{S2}$$

$$w_\infty(v) = \frac{1}{2}\left(1 + \tanh\left((v - v_C)/v_D\right)\right). \tag{S3}$$

Model parameters were adapted from Bose and Booth (2011) and are given in table S1:

Table S1. Default parameters for coupled Morris-Lecar model.

Parameter	value
g_{L}	0.15 mS/cm^2
g_{Ca}	0.3 mS/cm^2
g_{K}	0.6 mS/cm^2
v_{L}	-50 mV
v_{Ca}	100 mV
v_{K}	-70 mV
v_A	1 mV
v_B	14.5 mV
v_C	4 mV
v_D	15 mV
I	3.8 μA/cm^2
τ_w	100 ms
τ_a	1000 ms
τ_b	100 ms
τ_κ	100 ms
v_θ	0 mV
v_s	-80 mV
T	376 ms
T_a	49 ms
T_s	327 ms
$g^\star$	0.0068 mS/cm^2
g_{bif}	0.0038 mS/cm^2
$\bar{g}_s$	0.584 mS/cm^2
$\lambda := \exp(-T_a/\tau_b)$	0.612
$\rho := \exp(-T_s/\tau_a)$	0.721

2 COMPUTING BIFURCATION DIAGRAM NUMERICALLY

The bifurcation diagram of stable $n : n$ solutions of the two-cell network in fig. 3 is obtained numerically as follows: We initialise the coupling strength at parameter values associated with one type of $n : n$ solution, that is we choose the values $\bar{g} = 0.35, 0.4, 0.5, 0.52, 0.56$ for the $1 : 1$, $2 : 2$, $3 : 3$, $4 : 4$, and $5 : 5$ solutions respectively. For each $\bar{g}$ the system is then numerically integrated sufficiently long for any transients to fully subside. We then identify one period of the solution by finding the first return of the depression variable d_1. That is, we choose some value d_k at a spike time t_k, and by iterating from spike to spike find some subsequent value d_{k+1} at spike time such that $|d_{k+1} - d_k| < \epsilon$. If a periodic solution of type $n : n$ is found in such way, $\bar{g}$ is step-wise increased/decreased, and the above algorithm is repeated. Otherwise, the set of all previously found solutions and the corresponding values $\bar{g}$ are returned.

3 NUMERICAL VALIDATION OF CONSTANT ISI ASSUMPTION

To study the effect of the synaptic time constant τ_κ on consecutive ISIs of the active cell we consider a single cell that is inhibited by an exponentially decaying synaptic conductance g:

$$\dot{v} = f(v, w) - g(v - v_s), \tag{S4}$$

$$\dot{w} = h(v, w), \tag{S5}$$

$$\dot{g} = -g/\tau_\kappa, \tag{S6}$$

where functions f and h come from eqs. (3) and (4). We assume that the cell is released and fires its first spike at time $t = 0$. We therefore initialise v at the firing threshold v_θ, w at its nullcline, and the g at the release conductance $g^\star$, respectively:

$$v(0) = v_\theta, \tag{S7}$$

$$w(0) = v(0), \tag{S8}$$

$$g(0) = g^\star. \tag{S9}$$

We vary τ_κ and integrate the system numerically to record consecutive ISIs.

Figure S1 shows the curves for the first (ISI_1), second (ISI_2), and third (ISI_3) inter-spike-intervals for values $\tau_\kappa \in (0, 800]$. These results suggest that for $\tau_\kappa \leq 100$ we have $ISI_i \approx T$, that is, inhibition g decays sufficiently fast for its effect on the spiking period to be negligible. For $\tau_\kappa > 100$ the first ISI_1 becomes increasingly longer with τ_κ, moreover the effect of the inhibition propagates to the subsequent ISI_2 and ISI_3, suggesting that for a too slow synaptic time constant the assumption $ISI \approx T$ is not suitable.

REFERENCES

Bose A, Booth V. Co-existent activity patterns in inhibitory neuronal networks with short-term synaptic depression. Journal of Theoretical Biology 272 (2011) 42–54. doi:10.1016/j.jtbi.2010.12.001.

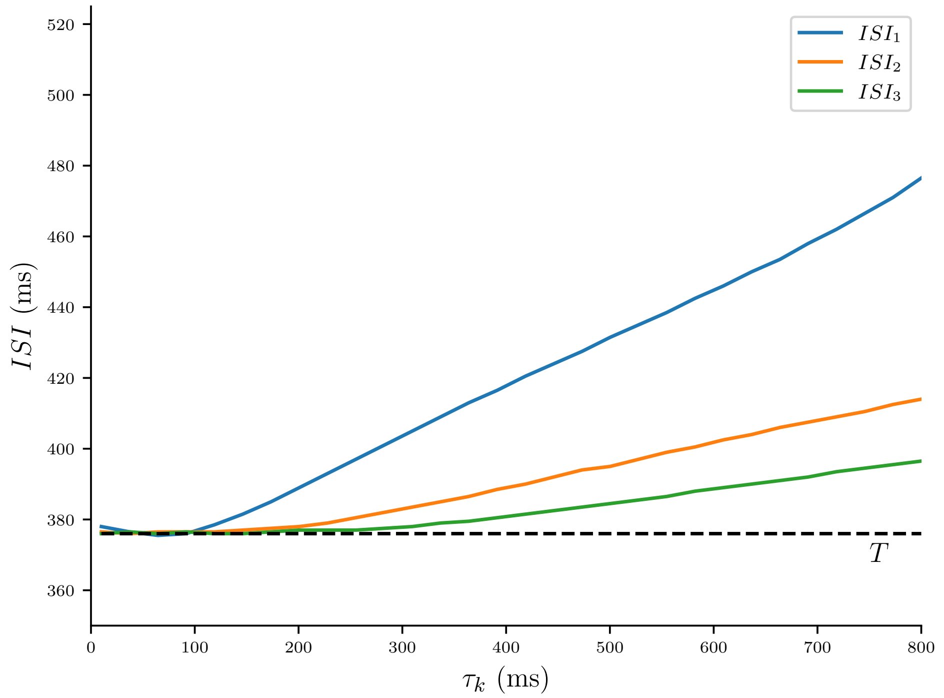

Figure S1. Numerically computed values of the first (ISI_1), second (ISI_2), and third (ISI_3) inter-spike-intervals as a function of $\tau_\kappa \in (0, 800]$.
